# Unemployment in Switzerland in the wake of the Covid-19 pandemic: an intertemporal perspective

**DOI:** 10.1186/s41937-020-00058-6

**Published:** 2020-08-18

**Authors:** George Sheldon

**Affiliations:** Economics Department, University of Basel, Basel, Switzerland

**Keywords:** Unemployment, Incidence, Duration, Covid-19, Switzerland

## Abstract

The following contribution compares the unemployment situation arising from the lockdown induced by the Covid-19 pandemic with previous employment crises in Switzerland. In addition, it forecasts the future trajectory of unemployment based on ongoing changes in hazard rates. From a historical perspective, current unemployment as well as that expected by the federal authorities in the medium term do not seem that dramatic. Current hazard rates present a different picture, however, predicting increases in both the unemployment rate and long-term unemployment to record levels.

## Introduction

The impact on the Swiss labor market of the lockdown that went into force on March 16, 2020, in the wake of the Covid-19 pandemic appears dramatic. Roughly 30,000 employed lost their jobs on average in March and April after the shutdown. In just those 2 months, the unemployment rate rose by almost as much as it increased in all of 2010 following the financial crisis. Accordingly, the State Secretariat for Economic Affairs currently expects the unemployment rate to average 4.1% in the coming year, eventually reaching 7% if the shutdown persists, and this despite the fact that over a quarter of the employed are presently working short time to avoid unemployment. Small wonder that some now fear that Switzerland will experience the deepest recession in its history in the coming months.

Yet is the current labor market situation really as dire or unique as presently felt? After all, roughly 30,000 individuals report unemployed every January without any major repercussions. Hence, strong surges in unemployment are not unheard of in Switzerland. The following contribution aims to enlarge upon this cursory evidence with a more in-depth study by comparing the current situation with previous employment crises and offering a forecast of the future trajectory of unemployment on the basis of a set of leading indicators that have proven their worth in the past.

Our results show that unemployment rates in excess of 4% are not unknown in Switzerland, the most recent occurrence arising in the 1990s. By decomposing the unemployment rates into its constituent flow components, unemployment incidence and duration, we further see that the lockdown has led to a new record with respect to incidence and almost equaled previous marks with regard to duration. In addition, forecasts of the future trajectory of the unemployment rate and the share of long-term unemployed based on the incidence and duration of unemployment prevailing in June 2020 imply that the unemployment rate will at least reach and perhaps even surpass the previous record set in the 1990s and that the share of unemployed will clearly exceed previous milestones.

## Historical perspective

**Fig. 1:**
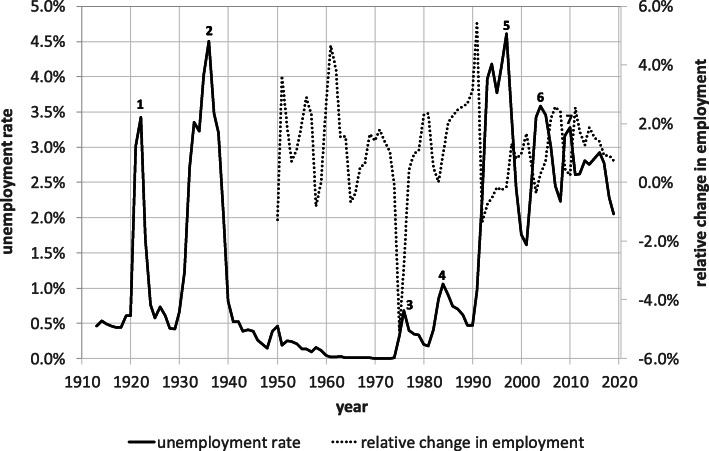
Employment and unemployment in Switzerland, annual averages, 1913-2019. 1—Great Deflation, 2—Great Depression, 3—1^st^ oil price shock, 4—2^nd^ oil price shock, 5—double-dip recession, 6—dotcom bubble burst, 7—financial crisis. Sources: Unemployment: 1913-1959: Table F. 18a, Swiss Economic and Social History Online; 1960-2019: Federal Statistical Office. Employment: 1913-1947: Table F. 18a, Swiss Economic and Social History Online; 1948-2019: Federal Statistical Office. Notes: The unemployment rate measures the registered share of the labor force (= unemployed + employed) unemployed at a given time, i.e., unemployed/(unemployed + employed). In contrast to official statistics of registered unemployment, which update the denominator of the unemployment rate at irregular intervals, the unemployment rates in the chart below rest on ongoing employment figures. Since employment in Switzerland exhibited a trend increase over the period viewed here, the unemployment rates in the figure may at times lie below official figures

Switzerland has experienced seven major employment downturns in the last roughly 100 years for which employment data are available (c.f., Fig. 1)^1^. The first occurred in the early 1920s when the Swiss National Bank resolved to break the high inflation stemming from an abandonment of the gold standard to finance the WWI-induced shortages and to return to the old gold standard. This led the inflation rate to drop from around +25% in 1918 to the today barely imaginable level of −20% in 1922. The rapid rate of deflation caused the economy to collapse and the unemployment rate to soar to 3.4%.

Years of relative prosperity followed thereafter before Switzerland underwent the global depression in 1931, which saw the average unemployment rate rise to 4.5% in 1936. The downturn did not last long, however, thanks in particular to the devaluation of the Swiss franc and the military build-up supported by a large bond issue in 1936.

After WWII, years of strong and broad growth followed, in which unemployment virtually disappeared. In fact, a general labor shortage arose leading to a strong influx of foreign workers, which in turn caused the share of foreign residents to rise from 6.1% in 1950 to 17.2% in 1970, a level last seen at the onset of WWI. However, it took 65 years to reach that level in 1914 but merely 20 years after the WWII.

The postwar years of prosperity came to an abrupt end with the first oil price shock in 1973/74 followed by the second one in 1982. Employment fell by almost 8% in the wake of the first shock, the largest job loss experienced by an OECD country in the wake of the first oil price crisis. Nevertheless, the unemployment rate did not even reach the 1% mark. One reason is that mandatory universal unemployment insurance did not exist in Switzerland until April 1977. As a result, roughly 80% of the labor force was not insured against unemployment at the time of the first oil price shock, and without benefit claims, many chose not to report their unemployment to the authorities and hence were not counted.

Another and quantitatively more important reason for the small rise in unemployment was the large share of foreign workers with temporary work permits that obligated them to exit Switzerland when they lost their jobs. Around 80% of job losses were absorbed in this way^2^. The proportion of foreigners with permanent residence permits has greatly increased since then, however, so that job losses today have a much greater impact on unemployment statistics than in the past.

Following the second oil price shock in the 1980s, the unemployment rate then did exceed 1%, and this, as Fig. 1 shows, despite the fact that merely the growth in employment and not its level declined. This is undoubtedly a consequence of the introduction of universal unemployment insurance in 1977, which requires registering at an employment office to draw benefits.

The next major downturn came in the early 1990s in the form of a double-dip recession. The first decline arose from a restrictive monetary policy aimed at combatting the high inflation stemming from an unintended monetary expansion resulting from the introduction of a new electronic interbank clearing system and from an overhaul of the liquidity requirements for Swiss banks. The second contraction in 1994/95, on the other hand, followed from a misreading of the business cycle by the Swiss National Bank and a subsequent over-restrictive monetary policy. Both shocks together caused the unemployment rate to rise to over 4.5%, a level last recorded during the Great Depression. This sharp rise is undoubtedly due in part to increased unemployment insurance coverage and the more sedentary foreign labor force.

The final two slowdowns came on the heels of the bursting of the dotcom bubble in the early 2000s and the global financial crisis in 2008/09, respectively. The reaction of employment to both downturns was rather subdued compared to the size of the changes in GDP, however. Econometric studies indeed confirm that the strength of the Okun relationship linking unemployment to the size and sign of the output gap has been steadily decreasing since 1990^3^. The reason is not yet clear but it may be due to the trend decline in production jobs that typically react more strongly to business cycle fluctuations.

Compared to the past, an unemployment rate of 4.1%, which the State Secretariat for Economic Affairs is expecting for 2021, thus does not seem that high or unique. Yet, as already mentioned, the limitations of the data presented in Fig. 1 are also to be taken into account. The unemployment data are of dubious quality prior to the introduction of mandatory unemployment insurance in April 1977. Moreover, annual employment data for the period before 1948 do not even exist. To calculate the unemployment rate, defined as the share of unemployed in the labor force, or the sum of employed and unemployed, for the period before 1948, one has to rely on the national censuses from 1910, 1920, 1930, and 1941, leaving large gaps for the intervening years. To make matters worse, the census employment data from those years exhibit a positive trend making it impossible to ascertain how many jobs were actually lost in the wake of the Great Deflation in the 1920s or during the Great Depression in the 1930s.

## Stocks and flows

The discussion above focused solely on the stock of unemployed and ignored the flows underlying stock changes. This is due to data availability. It was not until 1990 that the Swiss unemployment registration system was digitalized making it possible to study the separate contributions of layoffs and hires to changes in the stock of unemployed. Such knowledge is indispensable for forging effective labor market policy since most labor market measures are directed at flows. For example, short-time work attempts to stem inflows into unemployment while training measures aim to foster outflows into employment.

Two variables stand at the forefront of a stock-flow analysis of unemployment: the risk and the duration of unemployment. The risk of unemployment gives the probability that a member of the active labor force becomes unemployed in a given time interval and is calculated as the number of new entries into the stock of unemployed divided by the size of the labor force. The unemployment risk pertains to the incidence of unemployment. The duration of unemployment, on the other hand, measures the average length of a spell of unemployment, expressed in the same time units as the interval to which the risk of unemployment refers.

Measures of duration can apply to three types of unemployment spells: ongoing spells, beginning spells, and ending spells. The average duration of ongoing spells is probably the most widespread measure since its measurement only requires survey or cross-sectional data, which are commonly available. It has its shortcomings, however. For one, it only records the elapsed duration of unemployment at the time of the survey and hence is truncated. To measure the entire length of completed spells of unemployment requires panel data, which prior to the digitalization of unemployment registrations in 1990 were not regularly available in regard to unemployment in Switzerland.

The average elapsed duration of ongoing spells is not only subject to a truncation bias but to a sampling bias as well. The sampling bias stems from the fact that surveys sample from the stock of unemployed, which contains a greater share of long-term unemployment spells than found in the flows of beginning and ending spells. Which bias dominates empirically depends on the shape of the unemployment hazard function, which gives an individual’s probability of exiting unemployment as a function of the elapsed time already spent in that state. A decreasing hazard function means that the chances of escaping unemployment in the near future decline with the elapsed duration of unemployment. This is termed negative duration dependence and can result from scarring and/or unobserved heterogeneity among the unemployed^4^. If the hazard function is decreasing, the average duration of ongoing spells will exceed the average duration of completed spells despite being based on truncated spells^5^. This happens to be the case in Switzerland^6^.

Completed spells, on the other hand, can take two forms. They can pertain either to the spells ending or to those beginning in a particular time interval. The average duration of ending spells depends on the job opportunities that prevailed during the course of the spells it covers and thus is backward looking. As a consequence, the average duration of completed spells is typically higher in summer than in winter as numerous spells ending in summer begin in winter when job openings are rarer.

In the following, we focus on the duration of beginning spells of unemployment because it captures current instead of past job opportunities and thus better lends itself to the study of the evolution of job opportunities over time. However, unlike the other two duration measures, the duration of beginning spells cannot be observed directly but instead has to be calculated using probability theory^7^ and hazard rates. A hazard rate *h*(*t*) gives the probability that a jobless individual will exit unemployment after *t* periods of being unemployed. In turn, the difference [1 − *h*(*t*)], termed a survival rate, constitutes the probability of remaining unemployed in the same interval. In the following, we treat time as discrete and measure it in calendar months.

Under the assumption of stochastically independent hazard rates^8^, the following product

1 $$ S(k)=\prod \limits_{t=0}^k\left[1-h(t)\right] $$

equals the probability that an unemployment spell is still ongoing *k* months after its start. Since a time interval has a length of 1 month, (1) also gives the expected length of time, measured in fractions of a month, that an unemployed person will spend in the *k*th month of unemployment. *S*(.) is known as the survivor function, and *S*(*k*) gives the value of the function in the *k*th month.

Since (1) equals the expected time that an unemployed person will spend in the *k*th month of unemployment, summing (1) across all *T* + 1 duration intervals of length one, where *T* equals the longest spell length, yields the total expected time that the person will spend in unemployment or, equivalently, the expected duration of beginning spells^9^:

2 $$ \mathrm{expected}\ \mathrm{duration}=\sum \limits_{k=0}^TS(k)=\sum \limits_{k=0}^T\prod \limits_{t=0}^k\left[1-h(t)\right]. $$

As (2) indicates, the expected duration is equal to the area below the associated survivor curve. In demographic terms, (2) is equivalent to the life expectancy at birth, where the hazard rates represent age-specific mortality rates. In turn, the average duration of ongoing unemployment spells corresponds to the average age of the living, and the average duration of ending spells to the average age of the deceased.

Our procedure consists in calculating (2) for every calendar month using the observed monthly exit rates of the registered unemployed^10^ subdivided by elapsed duration as hazard rate estimates. Note that exits do not necessarily imply entering employment. This has important policy implications but has no bearing on the size of registered unemployment, which is the focus here.

As the estimated hazard rates used to calculate the expected duration of unemployment apply to a single calendar month, the ensuing duration measure reflects solely the job opportunities prevailing in the given month. This enables us to assign a spell duration to a single calendar month. This is both unique and of great practical use. It is unique because the average duration of unemployment is typically longer than a month and thus not attributable to an individual calendar month, and it is of great practical use because it enables the construction of a time series describing the evolution of job opportunities from month to month.

**Fig. 2:**
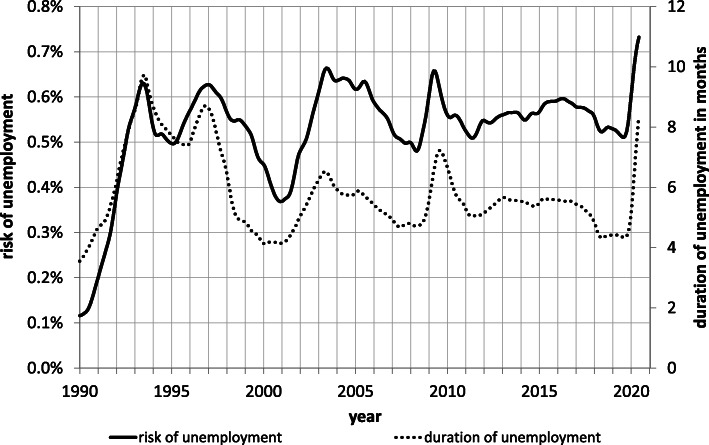
Risk and duration of unemployment, seasonally adjusted, January 1990-June 2020. Notes: The risk of unemployment measures the probability of becoming registered unemployed in a given calendar month and equals the number of individuals entering registered unemployment in that month divided by the current size of the labor force according to official registered unemployment statistics. The duration of unemployment gives the number of months that cohorts entering registered unemployment can expect to remain in this state and corresponds to the area under the survivor curve of the corresponding month calculated using Eq (2) and the computerized database underlying the official registered unemployment statistics

Figure 2 presents the seasonally adjusted monthly time series of the expected duration of beginning spells of unemployment for the months from January 1990 to June 2020 based on (2) along with the complementary series for the unemployment risk^11^. As a comparison with Fig. 1 indicates, the period covered in Fig. 2 includes three past employment downturns (the double-dip recession in the 1990s, the bursting of the dotcom bubble after 2000 and the financial crisis in 2008/09) as well as the current downswing caused by the lockdown in March 2020.

A number of things stand out in Fig. 2. For one, the chart reveals that the largest increases in both the risk and duration of unemployment occurred during the double-dip recession in the early 1990s. Switzerland has not experienced such large increases since. For another, it shows that the incidence and the duration of unemployment move roughly in unison across the business cycle suggesting that labor shedding and hiring freezes contribute approximately equally to cyclical upswings in unemployment.

Unique to the current crisis is the sharp upsurge in both the incidence and duration of unemployment. Never before have the two variables risen so quickly in such a short time span. This undoubtedly stems from the suddenness and the universality of the lockdown. Also striking is the rise in unemployment risk to a record level, especially in light of the widespread use of short-time work. Duration has not yet risen to record heights but may if the downturn persists.

## Future outlook

A major unknown at the moment is how the economy will fare in the coming months. Official statistics do not appear to provide much help in this regard. For example, preliminary estimates of GDP for the first quarter of 2020, which, experience shows, are thereafter subject to significant revisions, did not become available until early June and will not be accessible for the second quarter until early September, hardly in time to provide reliable guidance to economic policy.

In light of this, various researchers in Switzerland have begun searching for more up-to-date indicators of economic activity. Some have turned to transaction indicators such as credit card use or traveled miles, while others have focused on words searched in Google such as “unemployment” or “vacancies”. The time path of these indicators can give interesting insights into the general direction in which the economy is heading, but are not easily translated in terms of such major economic variables of interest as GDP or unemployment.

In the following, we present two leading indicators that avoid these shortcomings. They are timely, calculated monthly on the basis of the latest unemployment data that, unlike GDP estimates, are not subject to multiple revisions. In addition, the indicators are directly interpretable, have a good forecast record, and have been freely available to the public^12^ for over a decade.

Our indicators forecast the future values of (i) the unemployment rate and (ii) the share of long-term unemployed. The forecasts represent the values these variables would assume were the risk and expected duration of unemployment prevailing in a given calendar month to remain unchanged in the future. In other words, they correspond to the unemployment rate and the share of long-term unemployed that the risk and duration of unemployment prevailing in a given calendar month imply stochastically in a steady state, i.e., in the longer term.

Equation (1) serves as our point of departure in deriving the leading indicator for the unemployment rate. Since (1) gives the probability that an unemployment spell is still ongoing *k* months after its start, multiplying it with the size *n* of entering cohorts, as shown in (3), yields the expected number of individuals in the associated duration classes in steady state.

3 $$ n\cdot S(k)=n\cdot \prod \limits_{t=0}^k\left[1-h(t)\right] $$

Repeating the calculation in (3) for all *T* + 1 duration months or values of *k* and summing the results therefore yield the expected number of unemployed *U* that the size of entering cohorts and the expected unemployment duration prevailing in a given calendar month imply long term, i.e.,^13^

4 $$ U=n\cdot \sum \limits_{k=0}^TS(k)=n\cdot \sum \limits_{k=0}^T\prod \limits_{t=0}^k\left[1-h(t)\right] $$

Dividing both sides of (4) by the size *LF* of the labor force thus produces the corresponding unemployment rate *U*%:

5 $$ {\displaystyle \begin{array}{c}U\%=\frac{n}{LF}\cdot \sum \limits_{k=0}^TS(k)\\ {}\\ {}=\mathrm{risk}\times \left(\mathrm{expected}\ \mathrm{duration}\right)\end{array}} $$

Hence, by multiplying the two series in Fig. 2, one obtains a time series giving the expected future time path of the unemployment rate that the risk and the expected unemployment duration prevailing in the various months imply.

Note that (5), unlike most leading indicators, does not rest on an empirical regularity but, instead, on a mathematical law. Hence, a calculated expected unemployment rate would necessarily coincide exactly with the observed average long-term rate if the underlying unemployment risk and duration were to remain unchanged. As the latter is not to be expected, we update the indicator every month employing the latest data from the electronic unemployment registration system of the State Secretariat for Economic Affairs. This yields a rolling forecast of the future unemployment rate at monthly intervals.

**Fig. 3:**
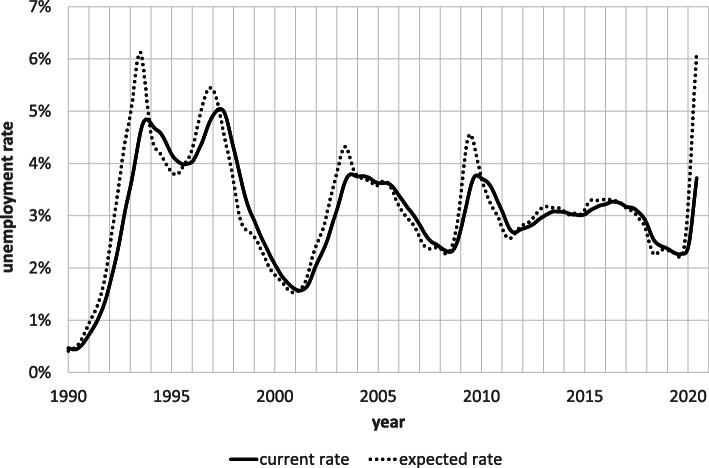
Current and expected unemployment rates, January 1990-June 2020, seasonally adjusted. Notes: The expected unemployment rate results from multiplying the risk and duration of unemployment appearing in Fig. 2 and in accordance to Eq (5)

A central issue at this juncture is the forecast accuracy of our indicator. Figure 3 provides a visual answer. As the chart shows, the relationship between the predicted (“expected rate”) and the observed unemployment rate (“current rate”) is much like that between marginal and average values. When our indicator (the marginal value) lies below the observed rate, the latter (the average value) falls, and when it lies above it, the latter rises. This finding implies that our leading indicator is a driving force underlying the time path of observed unemployment.

The acid test for any leading indicator is whether it succeeds in predicting the turning points in the series to which it pertains. Figure 3 suggests that that is the case in regard to our indicator. In fact, our indicator was the only one that in the fall of 2009 correctly predicted the trend reversal of the unemployment rate in 2010^14^. At the time, all forecasts predicted the unemployment rate, which stood just above 4% in late 2009, to exceed 5% in the coming year. In reality, it fell below 4%.

To determine the average lead time of our indicator, we search for the lagged value of the indicator that maximizes *R* squared in a regression of the observed unemployment rate on a single lagged value of the indicator. We find that *R* squared reaches its maximum of 97% at a lag length or lead time of 5 months. In Fig. 3, the lead time corresponds to the horizontal distance between the two curves.

What does our indicator portend now for the near future? The curves in Fig. 3 imply that, based on the risk and expected duration of unemployment prevailing in June 2020, the seasonally adjusted unemployment rate of 3.7% at the end of June will exceed 6% in 5 months, a level that the indicator last reached in the early 1990s. Note, though, that our indicator tends to overshoot the peaks in the published unemployment series by 0.5 to 1.0 percentage points. This is probably due to the fact that layoffs are usually quick and widespread, whereas recoveries are typically gradual and uneven. Consequently, the risk and expected unemployment duration vary asymmetrically across the cycle, increasing rapidly and strongly in a downturn, causing our indicator to rise sharply, and decreasing more gradually in an upswing, causing our indicator to decline slowly. Hence, our indicator may be providing an overly pessimistic forecast at the moment. Based on the experience of the 1990s, a forecast of 5% is probably more accurate. On the other hand, though, the indicator gives no indication of an approaching turnaround.

To derive our second leading indicator, the expected share *L* of long-term unemployed that current cohort sizes and expected unemployment durations imply for the future, note that the share of long-term unemployed is defined in Switzerland as the number of jobless that have been unemployed for at least 12 months divided by the total number of unemployed. Hence, based on (4), the expected share of long-term unemployed implied by the cohort size and expected duration of unemployment prevailing in a given calendar month equals

6 $$ L=\frac{n\cdot \sum \limits_{k=12}^TS(k)}{n\cdot \sum \limits_{k=0}^TS(k)}=\frac{\sum \limits_{k=12}^TS(k)}{\sum \limits_{k=0}^TS(k)}, $$

which is equivalent to the ratio of the area below the survivor curve above 11 months to the total area under the curve. As (6) indicates, the size of entering cohorts has no bearing on the value of the share of long-term unemployed. It depends solely on the slope of the survivor curve or the underlying hazard rates. Moreover, since it is essentially arbitrary where one draws the line between short and long-term unemployment, (6) implies more generally that the size of entering cohorts has no impact on the distribution of elapsed spell lengths in the steady-state stock of unemployed.

**Fig. 4:**
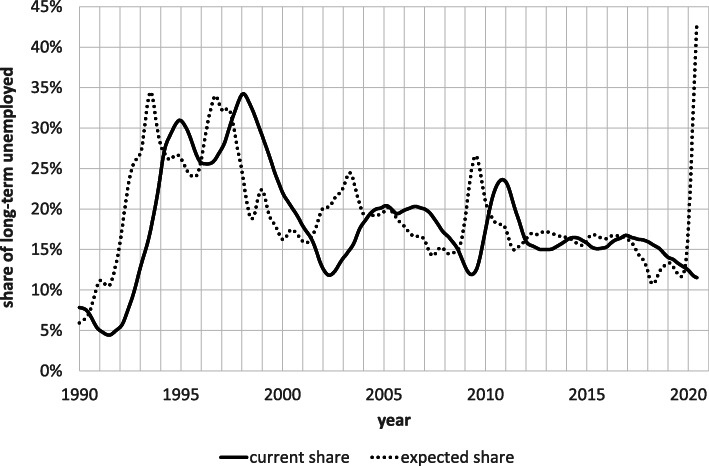
Current and expected share of long-term unemployed, January 1990-June 2020, seasonally adjusted. Notes: The expected share of long-term unemployed in a given month equals the ratio of the area under the survivor curve of that month above the 11-month mark divided by the entire area underneath the same curve according to Eq (6)

Figure 4 compares the time path of our leading indicator for the share of long-term unemployed (“expected share”) to that of the observed value (“current share”). As the chart indicates, our indicator exhibits a lead time of just over a year as one would expect given the definition of long-term unemployment. Regressions of the observed values of the share of long-term unemployed on varying lagged values of our leading indicator indicate that *R*-squared reaches its maximum of 91% at a forecast horizon of 16 months, which gives the average lead time of the indicator.

In light of its long forecast horizon, it is astonishing to observe in Fig. 4 how well the indicator predicts turning points in the observed share. We know of no other leading indicator that has this kind of forecast ability. Apparently, the die is cast so early in the unemployment process that the subsequent level of economic activity or policy interventions fails to have an impact on the share of long-term unemployed. Given this degree of persistence, Fig. 4 implies that the proportion of long-term unemployed, currently standing at 12%, will increase to the record level of over 40% in 16 months^15^, and this although the share of long-term unemployed is currently falling. However, the latter is merely a result of the current strong influx of new unemployment spells of short elapsed duration, which increases the denominator of the long-term unemployment share while leaving the numerator unchanged.

A large share of long-term unemployed is problematical. It tends to slow recovery because long-term unemployed, as declining hazard rates indicate, have greater difficulty finding jobs, be it that persistent unemployment signals serious deficiencies to potential employers, or be it that continuing unemployment destroys job skills. This can result in cyclical unemployment mutating into structural unemployment placing a large drag on recovery.

## Conclusion

Our discussion has shown that unemployment rates in excess of 4%, which the State Secretariat for Economic Affairs predicts for 2021, are historically not without precedent in Switzerland. Available statistics show that the unemployment rate rose above that level during the Great Depression in the 1930s and even as recently as in the early 1990s during the double-dip recession.

It is questionable, however, whether figures for registered unemployment collected prior to the introduction of mandatory unemployment insurance coverage in April 1977 lend themselves to comparison with today’s figures. Before the introduction of mandatory insurance, a large majority of the labor force (roughly 80% shortly before April 1977) was not insured against unemployment and, without claims to insurance benefits, probably did not report their unemployment to the authorities. Furthermore, the majority of foreign workers in Switzerland prior to the 1980s only held temporary work permits and had to exit Switzerland when they lost their jobs, further lowering the unemployment rate.

A decomposition of the unemployment rate into its flow components, unemployment incidence and duration, made possible by the digitalization of the federal unemployment registration system in 1990, indicates that increases in the two flow variables have contributed roughly equally to the sharp rise of the unemployment rate following the pandemic-induced lockdown in March. This has seen unemployment risk rise to a record height despite the fact that roughly a quarter of the labor force went on short-time work to avoid unemployment. The average duration of unemployment spells, on the other hand, remains just below its record length set during the double-dip recession in the early 1990s.

Our leading indicators for the unemployment rate and the share of long-term unemployed, which have yielded comparatively accurate forecasts in the past, point to a worsening labor market situation in the coming months. Based on current indicator values, we can expect the seasonally adjusted unemployment rate to climb from 3.7% at the end of June to roughly 5% in the next 5 months, and the proportion of long-term unemployed to rise from 12% currently to over 40% in the next 16 months. The forecast for the unemployment rate equals the previous record set in the early 1990s, while the forecast for long-term unemployment represents a new high mark. The strong increase in long-term unemployment is particularly worrisome as long-term unemployed are hard to place and hence put a drag on recovery.

In looking to the future, we expect labor shedding to taper off as the lockdown is eased and the duration of unemployment spells to lengthen as businesses enact hiring freezes in an attempt to climb out from under the mountain of debt they accumulated in lieu of VAT refunds to survive the lockdown. Lengthening unemployment spells will increase long-term unemployment for sure, but the effect of reduced incidence and increased duration on the unemployment rate is uncertain as the two forces counteract each other. The future will have to show which of the two effects proves to be decisive.

## Data Availability

The data presented in the paper were generated by the author from raw data drawn from the electronic unemployment registration system of the State Secretariat for Economic Affairs in Berne, Switzerland. The raw data are available under permission from the State Secretariat for Economic Affairs.

## Abbreviations

ALMP: Active labor market policy
Cf.: Confer/conferatur
GDP: Gross domestic product
OECD: Organisation for Economic Co-operation and Development
WWI: World War I
WWII: World War II

## Footnotes

1 For a more extensive discussion, see Baltensperger and Kugler (2017).

2 Cf. Lewin (1983).

3 Cf. Cueni and Sheldon (2013).

4 Cf. Heckman and Borjas (1980) for an extensive discussion of the phenomenon.

5 See Salant (1977).

6 Cf. https://wwz.unibas.ch/de/fruehindikatoren/

7 The mathematical theory of absorbing Markov chains developed by Kemeny and Snell (1960) forms the basis of our approach. Stone (1970, 1972, 1973) introduced Markov chains into economic literature. Sheldon (1996) first applied the concept to Swiss unemployment data. Sider (1985) and more recently Shimer (2012) have applied a similar approach to data from the US Current Population Survey (CPS). Their method is less straightforward, however, due to the survey quality of the CPS data.

8 Stochastic independence is a fundamental assumption of Markov chains.

9 Note that *h*(0) = 0 and *h*(*T*) = 1. We set *T* to 48 as very few unemployment spells in Switzerland last longer than 4 years.

10 Registered unemployed are defined in Switzerland as jobless aged 15-64 who are registered at an employment office on the last day of the month and are immediately available for work. Registered jobless who are not immediately available on the last day due, say, to participation in an active labor market policy (ALMP) program such as re-training are not counted as registered unemployed. They typically make up roughly a fifth of all people registered at an employment office. Counting them as unemployed would raise the unemployment rate by one percentage point on average. Another 5% of the registered consist of individuals who are not immediately available for work due, say, to illness, who are still within the notice period, or who are working yet receiving an insurance benefit such as reimbursement for commuting costs. The remaining 75% consist of registered unemployed.

11 Including participants in an ALMP program raises duration by 2 months and lowers risk by 0.02 percentage points on average, the latter due to the fact that some entries into registered unemployment represent returning participants from ALMP programs that are excluded when ALMP participants are counted as registered unemployed.

12 See https://wwz.unibas.ch/de/fruehindikatoren/ .

13 If hazard rates *h*(*t*) do not vary with elapsed time *t*, (4) reduces to 1/*h*, or 1/(1−*s*) in terms of the complementary uniform survivor rate *s*. The similarity to the Keynesian income multiplier is not just coincidental since both represent the sum of an infinite decreasing geometric series. Moreover, the similarity points out the fact that the expected spell duration acts like a multiplier in translating the flow *n* of new unemployment spells into the stock *U* of unemployed. The longer the duration, the larger is the stock of unemployed that a given cohort size produces. In the matrix notation of Markov chains, the reciprocal 1/(1−*s*) corresponds to the inverse matrix [***I***−***S***]^−1^, which is known as the fundamental matrix of a Markov chain and in input-output analysis as the Leontief inverse, where input coefficients replace the survival rates *s*(*t*) represented by ***S*** in the inverse matrix. The mathematics are the same, however, which is why contributions by Stone (1970, 1972) often appear in works on input-output analysis.

14 Cf. Sheldon (2009).

15 Counting the participants in ALMP programs as unemployed raises both shares by three percentage points on average.
